# The sarcoid granuloma: ‘epithelioid’ or ‘lymphocytic-epithelioid’ granuloma?

**DOI:** 10.1186/2049-6958-7-11

**Published:** 2012-06-20

**Authors:** Zdravko Kosjerina, Bojan Zaric, Dejan Vuckovic, Dusan Lalosevic, Goran Djenadic, Bruno Murer

**Affiliations:** 1 Pathology Department, Institute for Lung Diseases of Vojvodina, 21234, Sremska Kamenica, Serbia; 2 Bronchology Department, Institute for Lung Diseases of Vojvodina, 21234, Sremska Kamenica, Serbia; 3 Pasteur Institute, Hajduk Veljkova 1, Novi Sad, Serbia; 4 General Hospital, Sabac, Serbia; 5 Dell’Angelo Hospital, via Paccagnella 11, Mestre (Venice), Italy

**Keywords:** Epithelioid granuloma, Lymphocytic-epithelioid granuloma, Morphometry, Sarcoidosis

## Abstract

**Background:**

This study aims to analyze the structure and quantities of cellular elements in sarcoid granulomas.

**Methods:**

We investigated 34 transbronchial lung biopsy samples obtained from 34 sarcoid patients. The quantity and composition of the cellular elements inside a granuloma were determined by the quantitative stereometry method, employing the numerical density as a stereological method.

**Results:**

A total of 102 sarcoid granulomas were analyzed. The central part of all granulomas was occupied by epithelioid cells. Besides these, giant cells, lymphocytes, macrophages and plasma cells were also seen. The mean numerical density of all the cells in the central part of a sarcoid granuloma was 111,751 mm^-3.^ Lymphocytes prevailed in number, exceeding the total count of all other cells. With a mean numerical density of 74,321 mm^-3^, lymphocytes were twice as numerous as both epithelioid cells and macrophages with a mean numerical density of 37,193 mm^-3^.

**Conclusions:**

Lymphocytes are the predominant cell type in the central part of a sarcoid granuloma, significantly exceeding both epithelioid cells and macrophages in number, raising the question if the term “epithelioid granuloma”, routinely used to designate sarcoid granulomas, is correct, or if it would be more logical to call them “lymphocytic-epithelioid granulomas” instead.

**Trial registration:**

This study was supported by the Serbian Ministry of Science and Environmental Protection Grant Number 175006/2011.

## Background

Sarcoidosis is a multi-system disease of unknown etiology, usually affecting the respiratory tract and other organs, and is characterized by the formation of nonnecrotizing epithelioid granulomas [1]. Granulomas are structured masses composed of lymphocytes and macrophage-derived cells, which assume an epithelioid aspect [2,3]. They are composed of focal collections of macrophages and their derivatives, as well as of lymphocytes.

The central part of a granuloma is composed of macrophages, modified macrophages, epithelioid cells and giant cells, with scattered, predominately CD4+ T, lymphocytes between them [4]. The peripheral part of a granuloma is predominantly occupied by lymphocytes, fibroblasts, sparse macrophages and fibrocytes. CD4+ T lymphocytes predominate in the inner layer of the granuloma periphery and CD8+ T lymphocytes in the outer one [5]. B lymphocytes are usually very rare in a granuloma [6]. Caseous necrosis is absent, while central necrosis, as a granular acidophilic focus without nuclear detritus, may be found [7]. This focus usually produces a periodic acid Schiff (PAS) positive reaction, suggesting the fibrinoid necrosis is involved here.

The purpose of the investigation was to analyze the structure and quantities of cellular elements in sarcoid granulomas.

## Methods

The material of the investigation included 34 transbronchial lung biopsy samples obtained from 34 sarcoid patients - 22 (64.7%) females and 12 (35.3%) males, mean age 43.2 years. Each specimen was embedded in paraffin and five microns thick sections were stained with hematoxylin-eosin. All patients had stage II sarcoidosis. The diagnosis of sarcoidosis was based on histological, clinical and radiological evidence. The morphologic diagnosis of pulmonary sarcoidosis relies on three main findings: the presence of tight, well-formed granulomas with a rim of lymphocytes and fibroblasts in the outer margin; a perilymphatic interstitial distribution of the granulomas; and exclusion of an alternative cause [8].

High resolution computed tomography was used to diagnose stage (Stage II – bilateral hilar lymphadenopathy and diffuse pulmonary infiltrations).The granulomas were interactively divided into a central part and a peripheral part. The central part of the granuloma consists of sessile macrophages, which have been transformed into epithelioid cells, and by fusion form the multi-nucleated Langhans giant cells. In the periphery lie activated T-lymphocytes, predominantly CD4+ lymphocytes, some CD8+ lymphocytes and a few B lymphocytes [9].

The quantity and composition of the cellular elements inside a granuloma were determined by the quantitative stereometry method [10-12]. The multi-purpose Weibel's test system M42 was used as an instrument for stereometric analysis.

Numerical density was utilized in the study as a stereological method. The numerical density is a relative stereological variable denoting the number of particles in a space unit. The numerical density dimension is mm^-3^[13].

The numerical density of the particles is calculated by the formula:

(1) $$N_{v}=N_{A}/\left(DF+D\right)$$

in which N_A_ is the numerical areal density of the particles, DF represents the depth of focus, and D the mean diameter of the particles.

The depth of focus is calculated by the formula:

(2) $$DF=n^{*}4^{*}\lambda^{*}0.125/NA^{2}$$

in which “λ” represents the light wave length (550 nm), “n” is the refractive index of the specimen, i.e. practically of the mounting medium, and close to the index of glass, 1.52, and “NA” represents the numerical aperture of respective magnification lens [14].

## Results

In 34 transbronchial lung biopsy samples examined, a total of 102 sarcoid granulomas were found (1–11 in a biopsy sample). Three sarcoid granulomas per biopsy were registered on average. Most of the granulomas were solitary and only 7 were coalesced. In these 7 coalesced granulomas, a total of 20 solitary granulomas were found.

The mean numerical density of all the cells in the central part of sarcoid granulomas was 111,751 mm^-3^, ranging from 43,897 to 197,986 mm^-3^.

Lymphocytes prevailed in number, being double the number of both epithelioid cells and macrophages. This is a statistically significant difference (p < 0.05).

Both epithelioid cells and macrophages were exclusively found in the central region of all examined granulomas. Their numerical density ranged from 15,379 to 65,477 mm^-3^, or 37,193 mm^-3^ on average.

In addition to epithelioid cells, giant cells were also found in the central region of 63 (61.76%) granulomas, while 39 (38.23%) of the examined granulomas contained no giant cells.

Lymphocytes were observed in both the central and peripheral region of all examined granulomas. The numerical density of the lymphocytes localized in the central granuloma region ranged from 28,518 to 131,436 mm^-3^, with the mean numerical density of 74,321 mm^-3^.

Plasma cells were present in the centre and periphery of 6 (5.88%) and 9 (8.82%) examined granulomas respectively (Table 1).

**Table 1:** Cellular elements in the central region of sarcoid granuloma

Cell type	Mean count	%	Minimum	Maximum
Epithelioid cells and Macrophages	37,193	33.28	15,379	65,477
Giant cells	226	0.20	0	870
Lymphocytes	74,321	66.51	28,518	131,436
Plasma cells	11	0.01	0	203
Total	111,751	100.00	43,897	197,986

Numerical Density (in mm^-3^).

## Discussion

Our data show that the most prevalent population in the central part of sarcoid granuloma are lymphocytes, a rather unexpected finding supported by a new morphometric approach.

Analyzing a biopsy sample of a sarcoid patient, Soler and Basset [15] found mature epithelioid cells in the central granuloma region, terming it a follicle. He considered epithelioid cells to have been the main component of a sarcoid granuloma.

Carrington [16] examined 49 sarcoid samples and expressed the density of cell populations according to a semiquantitave score of density (0 - a normal finding; 10 - the most intensive change). He found epithelioid cells were the predominant component (6.0 degrees), followed by lymphocytes (5.0 degrees), and giant cells (4.8 degrees).

Ferluga [17] analyzed 14 bronchobiopsy sarcoid samples and established “the epithelioid cells as the predominant cellular component of sarcoid granulomas, except in the terminal, sclerotic stage of the disease”. Fusse et al. [18] found a great number of epithelioid cells in the central part of a sarcoid granuloma.

Basset et al. [19] reported that epithelioid cells were (at least regarding their quantity) the predominant component of sarcoid granulomas.

Cardoso et al. [20], in an analysis of 31 biopsy samples of cutaneous sarcoidosis, found epithelioid cells present in 100% of the cases, and multinucleate giant cells in 30 biopsies (97%). Lymphoid cells were also seen in all cases, but only in a small number in 22 biopsies (71%).

Rosen reported: “Lymphocytes are often numerous and are well visualized by light microscope in the peripheral cellular mantle of the sarcoid granuloma. Although lymphocytes are seldom conspicuous in the central portion of granuloma, they can be visualized readily by electron microscopy” [21].

All these authors have reported that epithelioid cells were the major or basic element of a sarcoid granuloma. A long list of other authors [22-56] have called sarcoid granuloma “epithelioid granuloma”, which means they acknowledge the fact that epithelioid cells represent the fundamental and most numerous cell element in sarcoid granuloma, while Carrington [16], Baset et al. [19] and Cardoso et al. [20] have explicitly stated that epithelioid cells predominated in quantity in a sarcoid granuloma.

This is not in correlation with our findings of lymphocytes as the predominating cells in the central part of a sarcoid granuloma, significantly exceeding the number of epithelioid cells.

**Figure 1:**
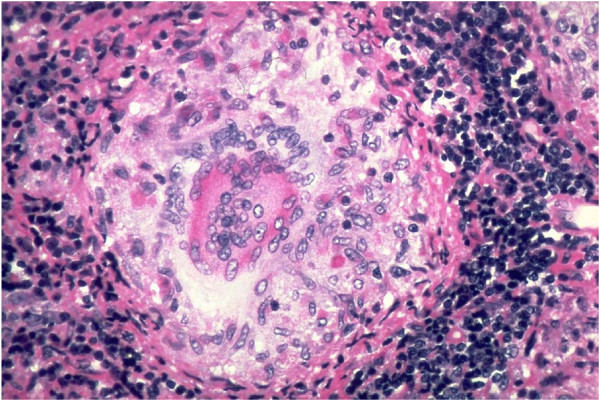
Sarcoid granuloma: “Epithelioid granuloma” or “Lymphocytic-epithelioid granuloma”.

What is this discrepancy due to? The reason may probably be found in the fact that the cited authors analyzed sarcoid granulomas by the semiquantitative method, producing the impression of predominating epithelioid cells as they occupied the largest area in the cross-section of a granuloma. However, the epithelioid cells in the central part of a sarcoid granuloma were significantly fewer in number than lymphocytes (Figure 1).

We would try to explain this by an image. Let us imagine a room with 10 ping-pong balls, as well as 10 large balls of 1 m in diameter, randomly thrown around. If we now make parallel imagined cross-sections from the ceiling to the floor at 2 m distance, according to the theory of probability, we would cut two or more large balls, and at the same time only one or none of the ping-pong balls. Analyzing now the obtained cross-sections, we would wrongly conclude that there are more large balls in the room, although the number of large and ping-pong balls in the room is in fact the same. Figuratively, ping pong balls stand for lymphocytes, large balls represent epithelioid cells and the imagined cross-sections of the room are histological cuts.

## Conclusions

1. Lymphocytes are the predominant cell type in the central part of a sarcoid granuloma, significantly exceeding both epithelioid cells and macrophages in number. This difference is statistically significant.

2. It is justifiable to question the term “epithelioid granulomas” which has been used to designate sarcoid granulomas. Would it not be preferable to term these granulomas “lymphocytic-epithelioid granulomas” instead? We fervently hope this question will be answered soon.

3. We are very well aware of the fact that it is not only the count of the cells, but also their function and role that are important, and all must be considered in the pathogenesis of sarcoidosis.

## Consent

Written informed consent was obtained from the patient for publication of 227 this report and any accompanying images.

## Competing interests

The authors declare that they have no competing interests.

## Authors’ information

Relating to Figure 1, courtesy of Dr Yale Rosen, SUNY Downstate Medical Center, Brooklyn, NY, USA.
